# Paracrine and endocrine modes of myostatin action

**DOI:** 10.1152/japplphysiol.00874.2015

**Published:** 2016-01-14

**Authors:** Yun-Sil Lee, Thanh V. Huynh, Se-Jin Lee

**Affiliations:** ^1^Department of Molecular Biology and Genetics, Johns Hopkins University School of Medicine, Baltimore, Maryland

**Keywords:** endocrine, myostatin, paracrine, skeletal muscle

## Abstract

Myostatin (MSTN) is a secreted signaling molecule that normally acts to limit muscle mass. In adult animals, MSTN is made almost exclusively by skeletal muscle and circulates in the blood. A critical question is whether this circulating MSTN protein can enter the active pool to regulate muscle growth or whether all of the activity of MSTN results from locally produced protein. Here, we addressed this question in mice by using a *Cdx2-Cre* transgene in conjunction with a conditional *Mstn-flox* allele to generate mice in which *Mstn* was targeted in a regionally restricted manner. Specifically, we generated mosaic mice in which MSTN production was eliminated in posteriorly located muscles but not in anteriorly located muscles, resulting in mice in which circulating levels of MSTN were reduced roughly by half. Analysis of posteriorly located vs. anteriorly located muscles of these mice revealed clear differential effects indicative of an important paracrine role for MSTN in regulating muscle mass. Significant, albeit more subtle, effects consistent with an endocrine mode of MSTN action were also seen in these mice. These findings have important implications not only for the understanding of the physiological control of muscle mass but also for therapeutic strategies to target MSTN to treat patients with muscle loss.

myostatin (MSTN) is a secreted growth and differentiation factor that belongs to the transforming growth factor-β (TGF-β) superfamily (19). Homozygous disruption of the *Mstn* gene in mice (*Mstn*^*−/−*^) causes dramatic increases in skeletal muscle mass throughout the body, suggesting that MSTN acts as a potent negative regulator of skeletal muscle mass (19). The function of MSTN is highly conserved among mammals, as naturally occurring mutations in the *MSTN* gene result in muscular hypertrophy in many different mammalian species, including cattle (6, 7, 11, 20), sheep (3), dogs (21), and humans (24). Pharmacological blockade of MSTN in adult mice can also cause significant increases in muscle growth (15), and as a result, there has been considerable effort directed at developing MSTN inhibitors to treat a wide range of disease states characterized by muscle loss.

MSTN appears to have at least two distinct functions in regulating muscle mass. During embryogenesis, *Mstn* is expressed in developing somites, which give rise to skeletal muscle, and acts to regulate the number of muscle fibers that are formed. Postnatally, *Mstn* is expressed almost exclusively in skeletal muscle and regulates growth of muscle fibers (19). MSTN is known to circulate in the blood, and a critical question is whether this circulating protein can enter the active pool to regulate muscle growth. In this respect, several studies have raised the possibility that increased levels of circulating MSTN protein may play a role in the etiology of cachexia. In particular, systemic overexpression of myostatin has been shown to induce profound muscle loss (26), and increased MSTN levels in serum have been observed in disease states such as cancer, acquired immunodeficiency syndrome, chronic kidney failure, and heart failure (5, 8, 9, 22). Moreover, *Mstn* expression in the heart has been shown to be upregulated following injury (1, 2), and one study reported that heart-specific deletion of *Mstn* could prevent the development of cardiac cachexia; that is, deletion of *Mstn* in cardiac muscle could prevent skeletal muscle atrophy in mouse models of heart failure (9). Although the results from this study were consistent with a systemic mode of action of MSTN in the setting of heart failure, direct evidence for a systemic mode of action of MSTN under normal physiological conditions has been lacking. Perhaps the best evidence for a systemic mode of action of MSTN was our finding that the *Mstn* loss-of-function mutation exerts a maternal effect on muscle mass of the offspring, such that genotypically identical offspring of mothers with fewer functional *Mstn* alleles exhibited greater muscle mass (16). Studies in which we transferred neonates at birth to mothers of different genotypes revealed that this maternal effect results entirely from effects during embryonic development, consistent with a key regulator being able to cross the placenta to regulate muscle mass in the embryo, the simplest possibility being that this key regulator is MSTN itself.

Here, we have further investigated the mode of action of MSTN by generating mosaic mice in which we targeted MSTN production in a regionally restricted manner. Specifically, we generated mice carrying a conditional *Mstn-flox* allele and then targeted recombination only in the posterior region of the animal using a *Cdx2-Cre* transgene, which is expressed only in tissues posterior to the umbilicus. By analyzing muscles in different regions of these mosaic mice, we observed effects consistent with both local and systemic modes of MSTN action.

## MATERIALS AND METHODS

### 

#### Mice.

**Fig. 1.**
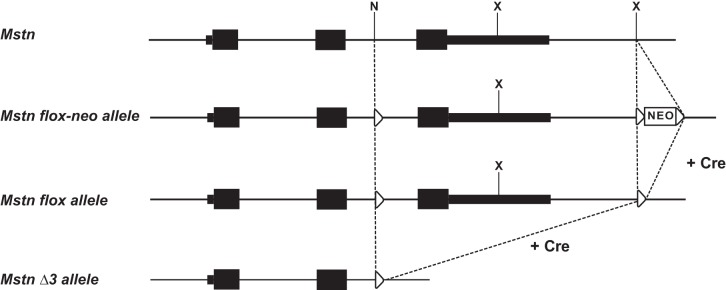
Gene targeting strategy for *Mstn* conditional knockout mice. The 3 exons are shaded in black; untranslated regions (UTRs) are shown as narrow boxes, and coding regions are shown as thicker boxes. In the targeting construct, we flanked exon 3 with LoxP sites (triangles); one LoxP was inserted in *Nde*I (N) site in intron 2 and two LoxPs flanking neomycin resistance cassette (NEO) were inserted in *Xba*I (X) site downstream of the 3′-UTR. *Cre*-mediated recombination of the LoxP sites flanking NEO results in an *Mstn*^*flox*^ allele. *Cre*-mediated recombination of the *Mstn*^*flox*^ allele generates the *Mstn*^*Δ3*^ allele.

*Mstn* straight knockout mice have been described previously (19). To generate *Mstn* conditional knockout mice, we generated targeting constructs in which we flanked exon 3 with LoxP sites (Fig. 1); because exon 3 contains the entire C-terminal domain, which is the active signaling portion of MSTN, it seemed almost certain that deletion of exon 3 by cre-mediated recombination would result in a null allele. *Cdx2-Cre* transgenic mice in the C57BL/6 genetic background (Stock No. 009350) were purchased from the Jackson Laboratory (Bar Harbor, ME). To analyze the effect of *Cdx2-Cre* in *Mstn*^*flox/flox*^ mice, *Cdx2-Cre* transgenic males were mated with *Mstn*^*flox/flox*^ females. *Cdx2-Cre;Mstn^flox/+^* males from this cross were mated to *Mstn*^*flox/flox*^ females to obtain *Cdx2-Cre;Mstn*^*flox/flox*^ males. Mice from the mating between *Cdx2-Cre;Mstn*^*flox/flox*^ males and *Mstn*^*flox/flox*^ females were analyzed. *Mstn*^*+/−*^ mice were generated from the mating between *Mstn*^*−/−*^ males and C57BL/6 females. All mice were maintained on a C57BL/6 background. Mice were handled and housed according to the approved Institutional Animal Care and Use Committee (IACUC) protocols MO13M283 and MO14M455 of the Johns Hopkins Medical Institutions. All animal studies were approved by the IACUC of the Johns Hopkins Medical Institutions.

#### Muscle weight and histological analysis.

For measurement of muscle weights, individual muscles from both sides of 10-wk-old mice were dissected, and the average weight was used for each muscle. The right triceps and gastrocnemius muscles were embedded in optimal cutting temperature (OCT) compound and snap frozen in isopentene cooled in liquid nitrogen. Ten-micrometer cross-sections taken from the frozen muscles were subjected to hematoxylin/eosin (H&E) and immunohistochemistry for laminin-2 (monoclonal anti-laminin-2 antibody produced in rat, Sigma-Aldrich, St. Louis, MO) to outline the muscle fibers. For morphometric analysis, fiber diameters in gastrocnemius muscles (4 mice per group) were measured as the shortest width passing through the center of the fiber. Measurements were carried out on 250 fibers randomly selected from five representative areas of each section to estimate overall mean fiber diameter. For plotting the distribution of fiber sizes, all data for a given genotype were pooled (250 fibers per one mouse in 4 different mice per group, 1,000 fibers per group). The number of fibers was counted by Image-Pro Premier 9.0 (Media Cybernetics, Rockville, MD) based on laminin-2 stained gastrocnemius muscles (4 mice per group). Images were acquired using a Zeiss AxioCam MRc5 microscope in combination with AxioVision 4.8 software.

#### mRNA analysis by real-time quantitative PCR.

Pectoralis, triceps, quadriceps, and gastrocnemius muscles were homogenized in TRIzol Reagent (Thermo Fisher Scientific, Waltham, MA), and RNA was isolated from the supernatant with the RNeasy Mini kit (Qiagen, Valencia, CA) according to the manufacturer's instructions. All samples were treated with RNase-free DNase set (Qiagen, Valencia, CA) to remove trace amounts of genomic DNA. Complementary DNA (cDNA) was generated from the extracted RNA with the High Capacity cDNA Reverse Transcription Kit (Thermo Fisher Scientific, Waltham, MA) and quantified by real-time PCR assays. Sequence-specific primer and TaqMan 6-FAM dye-labeled MGB probe sets for *Mstn* and 18S rRNA were purchased from Applied Biosystems (Thermo Fisher Scientific, Waltham, MA), and real-time PCR assays were performed in triplicate for each sample in 3 mice per group. The expression level of *Mstn* in the *Cdx2-Cre;Mstn^flox/flox^* mouse muscles was normalized to 18S rRNA, and then compared with levels in the *Mstn*^*flox/flox*^ mouse muscles. Relative quantitation of gene expression was determined by standard 2^−ΔΔCt^ calculations (17).

#### Measurement of serum myostatin.

Serum myostatin levels were measured with the enzyme-linked immunosorbent assay (ELISA) kit (catalog no. DGDF80) from R&D Systems (Minneapolis, MN). Briefly, the serum samples were activated by 1 N HCl for 10 min at room temperature and neutralized by 1.1 N NaOH/0.5 M HEPES. The activated serum samples were diluted 50-fold with provided dilution buffer and assayed in duplicate within 2 h. The optical density (OD) of each well was measured at 450 and 540 nm, and the OD values at 450 nm were corrected by the OD values at 540 nm. The standard curve was generated in Gen5 Data Analysis Software (BioTek Instruments, Winooski, VT) using the four-parameter logistic curve-fit, and the concentrations of myostatin in the serum samples were calculated based on the corrected OD values.

#### Statistical analysis.

All values are presented as means ± SE from at least three independent experiments unless otherwise stated. An unpaired two-tailed Student's *t-*test and one-way ANOVA followed by a Tukey's post hoc test were performed for statistical analyses with two groups and more than two groups, respectively. *P* < 0.05 was considered significant.

## RESULTS AND DISCUSSION

To distinguish local effects from systemic effects of MSTN action, we sought to eliminate MSTN production in certain muscles but not in others. Our general strategy was to use Cre-mediated recombination to target deletion of a conditional *Mstn* allele in a regionally restricted manner throughout the body. We first generated mice carrying a *Mstn*^*flox*^ allele. As shown in Fig. 1, we generated a targeting construct in which we flanked exon 3 of the *Mstn* gene with LoxP sites. Our rationale for this design was that exon 3 encodes the entire COOH-terminal domain, which is the active signaling portion of MSTN that is generated by proteolytic processing of the precursor protein. Hence, deletion of exon 3 would be expected to result in a null allele; in fact, the deletion allele generated by Cre-mediated recombination of the *Mstn*^*flox*^ allele would be virtually identical to the original *Mstn* deletion allele that we described previously (19). Following electroporation of the targeting construct into embryonic stem (ES) cells, ES cell colonies carrying the homologously targeted allele were injected into blastocysts, and mice generated from these blastocysts were bred to identify those exhibiting germ-line transmission of the targeted allele. Offspring from these matings were then bred with *EIIa-Cre* transgenic mice (13) to delete the neomycin resistance (NEO) cassette in the germ line. From these crosses, we obtained mice carrying a *Mstn*^*flox*^ allele lacking the NEO cassette.

**Fig. 2.**
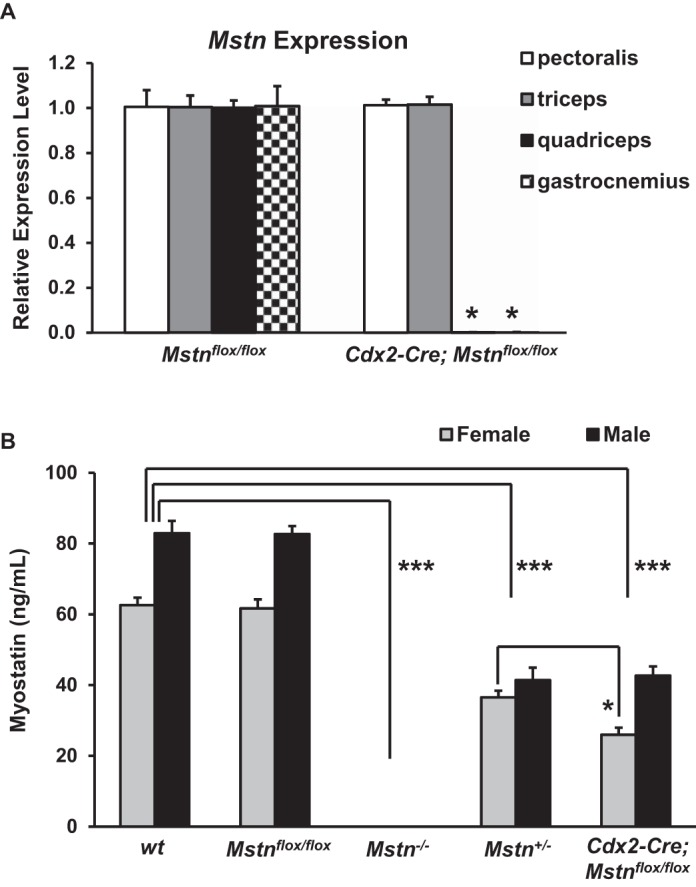
*Mstn* RNA expression and serum myostatin levels. *A*: *Mstn* RNA expression in pectoralis (white bar), triceps (gray bar), quadriceps (black bar), and gastrocnemius (checkered bar) muscles in *Mstn*^*flox/flox*^ and *Cdx2-Cre;Mstn*^*flox/flox*^ mice at 10 wk of age. *Mstn* RNA was not detected in the two posteriorly located muscles, quadriceps and gastrocnemius, of *Cdx2-Cre;Mstn*^*flox/flox*^ mice (*). Expression levels of *Mstn* in the *Cdx2-Cre;Mstn*^*flox/flox*^ mouse muscles were normalized to 18S rRNA and then compared with levels in muscles of *Mstn*^*flox/flox*^ mice. *B*: effect of *Cdx2-Cre* mediated conditional knockout of *Mstn* on serum myostatin levels. Note the dramatic decrease in circulating MSTN levels (ng/ml) by eliminating MSTN production in posteriorly located muscles using the *Cdx2-Cre* transgene (***P < 0.001 vs. wild type). Note the relative effect of *Cdx2-Cre* on MSTN serum levels was greater in females than in males; serum levels in *Cdx2-Cre;Mstn*^*flox/flox*^ female mice were reduced to levels significantly lower than those seen in *Mstn*^*+/−*^ female mice (*P < 0.05 vs. *Mstn*^*+/−*^). Data are shown as means ± SE.

To investigate local vs. systemic effects of MSTN loss, we crossed mice carrying the *Mstn*^*flox*^ allele to *Cdx2-Cre* transgenic mice. Because the *Cdx2-Cre* transgene is expressed in all solid tissues posterior to the umbilicus but not in tissues anterior to the umbilicus (10), we reasoned that using this approach, we would generate mice in which *Mstn* is expressed in anteriorly located muscles but not in posteriorly located muscles. From these crosses, we obtained *Mstn*^*flox/flox*^ mice with and without the *Cdx2-Cre* transgene. To confirm that the presence of the *Cdx2-Cre* transgene resulted in deletion of the *Mstn* gene in a regionally restricted manner, we performed real-time quantitative PCR to measure *Mstn* expression in four different muscles (pectoralis, triceps, quadriceps, and gastrocnemius) in *Mstn*^*flox/flox*^ and *Cdx2-Cre;Mstn*^*flox/flox*^ mice at 10 wk of age. As expected, *Mstn* RNA was not detected in the two posteriorly located muscles (quadriceps and gastrocnemius) of *Cdx2-Cre;Mstn*^*flox/flox*^ mice (Fig. 2*A*). In contrast, *Mstn* RNA levels in the two anteriorly located muscles (pectoralis and triceps) were indistinguishable in *Mstn*^*flox/flox*^ mice with and without the *Cdx2-Cre* transgene.

MSTN protein made by skeletal muscle is known to circulate in the blood. Because MSTN production would be eliminated in roughly the posterior half of the body in *Cdx2-Cre;Mstn*^*flox/flox*^ mice, circulating levels of MSTN would be predicted to be lower in *Cdx2-Cre;Mstn*^*flox/flox*^ mice compared with *Mstn*^*flox/flox*^ mice. To determine whether this is the case, we measured circulating levels of MSTN in 10-wk-old mice using a commercially available MSTN ELISA kit (R&D Systems). As shown in Fig. 2*B*, MSTN protein was readily detected using this assay in serum of wild-type (*wt*) mice, with circulating levels being measured at 62.6 ± 2.1 and 82.9 ± 3.5 ng/ml in females (*n* = 5) and males (*n* = 5), respectively. The specificity of this assay was confirmed by analysis of serum samples taken from mice homozygous for the original *Mstn* deletion allele (*Mstn*^*−/−*^), which showed no signal above background. Moreover, mice heterozygous for the *Mstn* deletion allele (*Mstn*^*+/−*^) exhibited intermediate MSTN serum levels of 36.5 ± 1.9 and 41.4 ± 3.5 ng/ml in females (*n* = 5) and males (*n* = 5), respectively, which were roughly half the levels seen in *wt* mice.

The presence of the LoxP sites in the *Mstn*^*flox*^ allele appeared to have little or no effect on *Mstn* expression, as circulating MSTN levels of *Mstn*^*flox/flox*^ mice were indistinguishable from those of *wt* mice. As expected, however, elimination of MSTN production in posteriorly located muscles using the *Cdx2-Cre* transgene had a significant effect on circulating MSTN levels. As shown in Fig. 2*B*, MSTN serum levels in *Cdx2-Cre;Mstn*^*flox/flox*^ mice were reduced to 26.0 ± 2.0 and 42.7 ± 2.6 ng/ml in females (*n* = 5) and males (*n* = 5), respectively. Of note, the relative effect of *Cdx2-Cre* on MSTN serum levels was greater in females than in males; that is, although the presence of the *Cdx2-Cre* transgene in males caused MSTN serum levels to be reduced approximately to those seen in *Mstn*^*+/−*^ mice, this effect was more pronounced in females, with serum levels being reduced to levels significantly lower than those seen in *Mstn*^*+/−*^ mice.

**Fig. 3.**
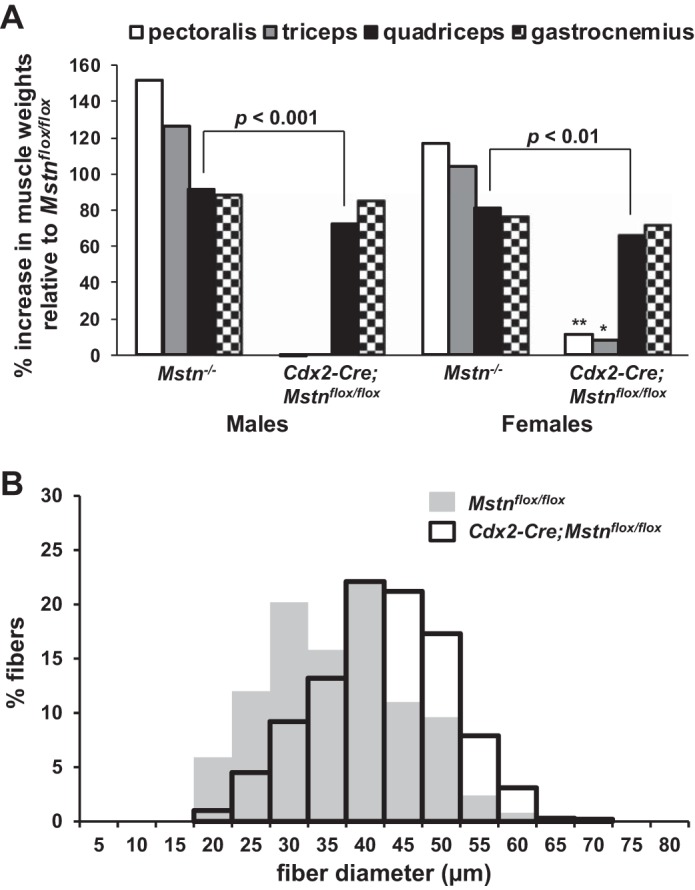
Effect of *Cdx2-Cre* on muscle weights and fiber size in *Mstn*^*flox/flox*^ mice at 10 wk of age. *A*: effect of *Cdx2-Cre* on weights of pectoralis, triceps, quadriceps, and gastrocnemius muscles in *Mstn*^*flox/flox*^ mice. Bars indicate percent increase in muscle weights in *Mstn*^*−/−*^ and *Cdx2-Cre;Mstn*^*flox/flox*^ mice compared with *Mstn*^*flox/flox*^ mice. Note the dramatic increase of weights of posteriorly located muscles (quadriceps and gastrocnemius) in both *Cdx2-Cre;Mstn*^*flox/flox*^ mice males and females: this effect was qualitatively similar to that seen in *Mstn*^*−/−*^ mice. Although the weight of the quadriceps muscle in *Cdx2-Cre;Mstn*^*flox/flox*^ mice was dramatically increased compared with *Mstn*^*flox/flox*^ mice, these increases were not as large as those seen in *Mstn*^*−/−*^ mice (*P* < 0.001 in male and *P* < 0.01 in female). In the case of anteriorly located muscles, both the pectoralis and triceps muscles of *Cdx2-Cre;Mstn*^*flox/flox*^ female mice were slightly increased in size (11.6% and 8.6%, respectively) compared with those of *Mstn*^*flox/flox*^ mice (*P* < 0.01 and *P* < 0.05, respectively). Data on muscle weights of *Mstn*^*−/−*^ mice were taken from Ref. 16. *P < 0.05 vs. *Mstn*^*flox/flox*^; **P < 0.01 vs. *Mstn*^*flox/flox*^. *B*: muscle fiber size distribution in *Mstn*^*flox/flox*^ (shaded bars) and *Cdx2-Cre;Mstn*^*flox/flox*^ (open bars) mice. Smallest cross-sectional fiber widths were measured in gastrocnemius muscles, and fiber sizes were plotted as a percent of total fiber number (1,000 fibers per group). The mean fiber sizes were 36.3 ± 1.4 μm for *Mstn*^*flox/flox*^ mice (*n* = 4) and 42.3 ± 1.0 μm for *Cdx2-Cre;Mstn*^*flox/flox*^ mice (*n* = 4).

**Fig. 4.**
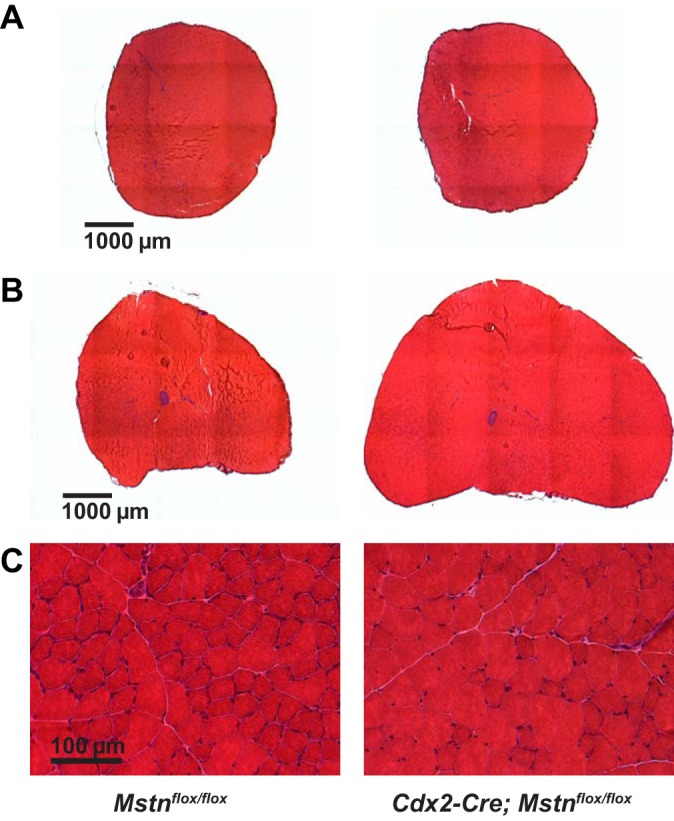
Differential effect of targeting the *Mstn* gene using the *Cdx2-Cre* transgene on posteriorly located vs. anteriorly located muscles. Sectioned triceps and gastrocnemius muscles were stained with hematoxylin and eosin (H&E). Note that the dramatic hypertrophy seen in the gastrocnemius muscle of *Cdx2-Cre;Mstn*^*flox/flox*^ mice (*B*) was not evident in the triceps muscle (*A*). Gastrocnemius muscles of *Mstn*^*flox/flox*^ mice and *Cdx2-Cre;Mstn*^*flox/flox*^ mice at higher magnification are shown in *C*.

Hence, by using the *Cdx2-Cre* transgene in combination with the *Mstn*^*flox*^ allele, we generated mice in which we were able to eliminate MSTN production in posteriorly located muscles without affecting MSTN production in anteriorly located muscles, thereby resulting in mosaic mice in which circulating levels of MSTN were significantly reduced. To assess the relative effects of local vs. systemic modes of MSTN action, we analyzed wet weights of four muscles (pectoralis, triceps, quadriceps, and gastrocnemius) in *Mstn*^*−/−*^, *Mstn*^*flox/flox*^, and *Cdx2-Cre;Mstn*^*flox/flox*^ mice at 10 wk of age. As shown in Fig. 3*A* and Table 1, weights of posteriorly located muscles (quadriceps and gastrocnemius) were dramatically increased in *Cdx2-Cre;Mstn*^*flox/flox*^ mice compared with *Mstn*^*flox/flox*^ mice; this effect was seen in both males and females and was qualitatively similar to that seen in *Mstn*^*−/−*^ mice. The dramatic increase in muscle size in *Cdx2-Cre;Mstn*^*flox/flox*^ mice was also clearly evident upon histological analysis of the gastrocnemius muscle (Fig. 4).

**Table 1.** Effect of Cdx2-Cre on muscle weights of Mstn^flox/flox^ mice

	*n*	Pectoralis, mg	Triceps, mg	Quadriceps, mg	Gastrocnemius, mg
Males					
*Mstn*^*flox/flox*^	17	76.8 ± 1.9	106.4 ± 2.7	207.1 ± 4.6	147.1 ± 3.6
*Cdx2-Cre;Mstn*^*flox/flox*^	22	76.6 ± 2.2^b^	108.0 ± 2.8^b^	357.2 ± 7.0^a^^,^^b^	272.1 ± 5.7^a^
*Mstn*^*−/−*^	17	193.7 ± 3.6^a^	240.7 ± 3.3^a^	397.3 ± 5.6^a^	277.7 ± 4.1^a^
Females					
*Mstn*^*flox/flox*^	22	51.0 ± 1.1	76.6 ± 1.8	153.8 ± 4.1	109.0 ± 2.5
*Cdx2-Cre;Mstn*^*flox/flox*^	15	56.9 ± 1.6^b^^,^^c^	83.2 ± 1.9^b^^,^^d^	256.1 ± 6.3^a^^,^^e^	187.5 ± 3.8^a^
*Mstn*^*−/−*^	19	110.6 ± 1.9^a^	156.9 ± 3.1^a^	278.6 ± 4.9^a^	192.5 ± 3.6^a^

Values are means ± SE. Data on muscle weights of *Mstn*^*−/−*^ mice were taken from Ref. 16.

a *P* < 0.001 vs. *Mstn*^*flox/flox*^;

b *P* < 0.001 vs. *Mstn*^*−/−*^;

c *P* < 0.01 vs. *Mstn*^*flox/flox*^;

d *P* < 0.05 vs. *Mstn*^*flox/flox*^;

e *P* < 0.01 vs. *Mstn*^*−/−*^.

To determine whether the increases in muscle mass resulted from hyperplasia or from hypertrophy, we counted the total number of fibers and measured fiber sizes in the gastrocnemius muscle. As shown in Table 2, the total number of muscle fibers was increased in gastrocnemius muscles of female *Cdx2-Cre;Mstn*^*flox/flox*^ mice (13,079 ± 471, *n* = 4) compared with those of *Mstn*^*flox/flox*^ mice (8,802 ± 337, *n* = 4), indicating that a large part of the increase in skeletal muscle mass resulted from muscle fiber hyperplasia. Muscle fiber hypertrophy also appeared to contribute to the overall increase in muscle mass. As shown in Fig. 3*B*, muscle fiber size distribution in *Cdx2-Cre;Mstn*^*flox/flox*^ mouse gastrocnemius muscles was shifted toward larger diameters compared with that in *Mstn*^*flox/flox*^ mouse muscles, with the mean fiber diameter being 16.5% larger in *Cdx2-Cre;Mstn*^*flox/flox*^ mice (42.3 ± 1.0 μm, *n* = 4) compared with *Mstn*^*flox/flox*^ mice (36.3 ± 1.4 μm, *n* = 4) (Table 2); assuming muscle fibers to be roughly cylindrical in shape, this increase in fiber diameter would be predicted to result in an increase in fiber cross-sectional area by ∼36%.

**Table 2.** Effect of Cdx2-Cre on the fiber number and diameter in Mstn^flox/flox^ mouse gastrocnemius muscle

	*n*	Fiber Number	Fiber Diameter, μm
*Mstn*^*flox/flox*^	4	8,802 ± 337	36.3 ± 1.4
*Cdx2-Cre;Mstn*^*flox/flox*^	4	13,079 ± 471^a^	42.3 ± 1.0^b^

Values are means ± SE.

a *P* < 0.001 vs. *Mstn*^*flox/flox*^;

b *P* < 0.05 vs. *Mstn*^*flox/flox*^.

These dramatic increases in muscle sizes that we observed in posteriorly located muscles were not seen in anteriorly located muscles (pectoralis and triceps) (Fig. 3*A* and Table 1). Hence, there was a striking difference between posteriorly located muscles and anteriorly located muscles in terms of the effect of targeting the *Mstn* gene using the *Cdx2-Cre* transgene, indicating that locally produced MSTN protein plays a critical role in regulating muscle mass. These mice, however, also exhibited a more subtle phenotype consistent with an endocrine role for MSTN in regulating muscle mass. Effects suggestive of a systemic role for MSTN were seen in both posteriorly located and anteriorly located muscles. In the case of posteriorly located muscles, the most significant effect was seen in the quadriceps muscle. In particular, although the weight of the quadriceps muscle in *Cdx2-Cre;Mstn*^*flox/flox*^ mice was dramatically increased compared with *Mstn*^*flox/flox*^ mice, these increases were not as large as those seen in *Mstn*^*−/−*^ mice. These differences in the weights of the quadriceps muscle between *Cdx2-Cre;Mstn*^*flox/flox*^ mice and *Mstn*^*−/−*^ mice were slightly greater in males compared with females and were highly statistically significant in both (*P* < 0.001 and *P* < 0.01, respectively). Because MSTN production was completely eliminated in quadriceps muscles of *Cdx2-Cre;Mstn*^*flox/flox*^ mice, the simplest interpretation of these data is that quadriceps weights in these mice were reduced by the action of circulating MSTN protein made by anteriorly located muscles. In the case of anteriorly located muscles, we observed the reverse effect, but in this case, the effect was seen only in female mice. In particular, both the pectoralis and triceps muscles of *Cdx2-Cre;Mstn*^*flox/flox*^ mice were slightly increased in size (11.6% and 8.6%, respectively) compared with those of *Mstn*^*flox/flox*^ mice (*P* < 0.01 and *P* < 0.05, respectively). Because levels of *Mstn* expression in the pectoralis and triceps muscles were indistinguishable between *Cdx2-Cre;Mstn*^*flox/flox*^ mice and *Mstn*^*flox/flox*^ mice, the simplest interpretation of these data is that muscle weights in the pectoralis and triceps were increased in *Cdx2-Cre;Mstn*^*flox/flox*^ mice as a result of the decreased circulating pool of MSTN protein resulting from elimination of MSTN production from the posterior region of the body.

Hence, our overall conclusion from these studies is that MSTN appears to regulate muscle mass using both paracrine and endocrine modes of action. The fact that there was a dramatic difference between effects of *Cdx2-Cre* mediated deletion of *Mstn* in the posterior region of the body on weights of posteriorly located muscles vs. anteriorly located muscles clearly shows that locally produced MSTN protein plays an important role in regulating muscle mass. MSTN also appears to have a systemic mode of action, however, as additional effects were seen in both posteriorly located muscles and anteriorly located muscles that could not be explained if MSTN acted only locally. In the case of posteriorly located quadriceps muscle, we saw a reduced effect of MSTN loss in *Cdx2-Cre;Mstn*^*flox/flox*^ mice compared with *Mstn*^*−/−*^ mice, suggesting partial rescue from circulating MSTN derived from the anterior region of the body. In the case of anteriorly located pectoralis and triceps muscles, muscle weights were increased in *Cdx2-Cre;Mstn*^*flox/flox*^ mice compared with *Mstn*^*flox/flox*^ mice even though *Mstn* expression in these muscles was unaffected, suggesting that the reduced circulating pool resulting from loss of *Mstn* expression in posterior regions led to reduced MSTN activity in anteriorly located muscles. Interestingly, we observed slight differences in these effects between males and females. The effect on the posteriorly located quadriceps muscle was more pronounced in males, whereas the effect on the anteriorly located pectoralis and triceps muscles was seen only in females. Although additional studies are required to confirm the significance of these sex differences, our findings were consistent with the slight difference that we observed on the effect of *Cdx2-Cre* on circulating levels of MSTN protein in males vs. females. Specifically, we found that circulating levels of MSTN protein in *Cdx2-Cre;Mstn*^*flox/flox*^ mice were reduced more significantly in female mice than in male mice. If circulating MSTN does play an important role in regulating muscle mass, this sex difference would be predicted to lead generally to higher relative muscle weights in female *Cdx2-Cre;Mstn*^*flox/flox*^ mice (relative to female *Mstn*^*flox/flox*^ mice) than in male mice. Indeed, we observed that the increases in quadriceps weights of *Cdx2-Cre;Mstn*^*flox/flox*^ mice compared with *Mstn*^*−/−*^ mice were less pronounced in males than in females, whereas the increases in pectoralis and triceps weights of *Cdx2-Cre;Mstn*^*flox/flox*^ mice compared with *Mstn*^*flox/flox*^ mice were more pronounced in females than in males.

Although these data suggest that both local and systemic modes of action of MSTN are significant, their relative significance in regulating muscle fiber growth in adult mice is difficult to tease out from these studies. In particular, MSTN is known to regulate muscle mass both during embryonic development by regulating the number of muscle fibers that are formed and postnatally by regulating muscle fiber growth. The approach used in our study would affect both roles of MSTN, and in this respect, we observed an increased number of muscle fibers in the gastrocnemius muscles of *Cdx2-Cre;Mstn*^*flox/flox*^ mice compared with *Mstn*^*flox/flox*^ mice, reflecting a significant contribution of a developmental effect on the overall phenotype. We presume that most if not all of the developmental effect reflects the paracrine role of MSTN; that is, given that fiber numbers are remarkably consistent among mice of a given strain, it seems likely that regulation of fiber numbers during embryogenesis would be relatively hard-wired through local effects of MSTN.

In contrast, fiber sizes can be influenced greatly in adult animals by a variety of physiological stimuli and can therefore vary widely not only from animal to animal but also in a given animal under different physiological conditions. A critical question has been whether MSTN functions at all in a systemic manner to regulate muscle growth, and several previous studies have implicated a role for circulating MSTN protein. Specifically, these studies have shown that systemic overexpression of myostatin in adult mice can cause significant muscle loss throughout the body (26), that heart-specific deletion of *Mstn* can prevent cardiac cachexia, that is, the loss of skeletal muscle mass in the setting of heart failure (9), and that the *Mstn* loss-of-function mutation exerts a maternal effect in affecting muscle mass of the developing embryo (16). Here, we have provided additional evidence that MSTN acts in an endocrine manner to regulate muscle mass. Although the effects that we observed in these studies were relatively subtle, for several reasons, we do not believe that these results necessarily imply that the endocrine role for MSTN is minimal. First, any effects that we observed in terms of postnatal regulation of muscle growth were in the background of a very substantial developmental effect on fiber numbers. Only by eliminating this developmental effect would it be possible to know the true relative importance of local vs. systemic modes of action in regulation of muscle fiber growth in adult mice. Second, inherent in our experimental design is the fact that the effects of local mode of action would be much more pronounced compared with those of systemic mode of action. Specifically, our experimental design led to complete loss of local production of MSTN in posterior regions, leading to elimination of paracrine signaling; as a result, with respect to local mode of action, this strategy would lead to a null phenotype. In contrast, our experimental design led to only partial loss (roughly half) of circulating MSTN protein; as a result, with respect to systemic mode of action, this strategy would lead to phenotypes roughly equivalent to those seen in heterozygotes, which are substantially less than those seen in homozygous mutants.

We believe that elucidating the role of circulating MSTN in regulating muscle growth is important for several reasons. First, if circulating MSTN does play an important role, the implication is that MSTN produced by one muscle can influence the growth of a distant muscle, which raises many questions regarding the physiological control of muscle mass. In this respect, we speculated previously that the size of the circulating MSTN pool may be critical in regulating the overall metabolic balance between fat and muscle throughout the body (14). Indeed, numerous studies have examined levels of MSTN in blood under a variety of physiological conditions, and such studies would have relevance only if the circulating MSTN protein can enter the active pool. Second, there is an enormous effort being undertaken to develop drugs to target MSTN for clinical applications, and there are currently at least 12 phase II clinical trials underway testing MSTN inhibitors in a wide range of indications. Major questions in this regard are whether targeting the circulating pool of MSTN will have therapeutic relevance and whether measuring effects of these therapeutic interventions on circulating MSTN levels will correlate at all with clinical efficacy. Finally, several recent studies have implicated an important role for GDF-11, a protein highly related to MSTN, in tissue aging. Specifically, GDF-11 circulating levels were reported to decline during aging (18), and restoration of GDF-11 levels by direct injection of purified GDF-11 to mice rescued age-related tissue dysfunction in the heart (18), brain (12), and skeletal muscle (25). Although the validity of some of these findings have been challenged by more recent studies (4, 23), understanding the role of circulating MSTN and GDF-11 will be critical to understanding the physiology of these molecules, particularly given that MSTN and GDF-11 are indistinguishable in terms of their in vitro activities and that many of the MSTN inhibitors in clinical development are capable of blocking both MSTN and GDF-11.

## GRANTS

This work was supported by the National Institutes of Health Grants R01-AR-059685, R01-AR-060636, and P01-NS-0720027 to S.-J. Lee. S.-J. Lee was supported by Lawrence Ellison Foundation Senior Scholar in Aging Award AG-SS-2678-11 and by generous gifts from Michael and Ann Hankin, Partners of Brown Advisory, and James and Julieta Higgins.

## DISCLOSURES

No conflicts of interest, financial or otherwise, are declared by the author(s).

## AUTHOR CONTRIBUTIONS

Author contributions: Y.-S.L., T.V.H., and S.-J.L. conception and design of research; Y.-S.L., T.V.H., and S.-J.L. performed experiments; Y.-S.L. and S.-J.L. analyzed data; Y.-S.L. and S.-J.L. interpreted results of experiments; Y.-S.L. prepared figures; Y.-S.L. and S.-J.L. drafted manuscript; Y.-S.L. and S.-J.L. edited and revised manuscript; Y.-S.L., T.V.H., and S.-J.L. approved final version of manuscript.
